# Sulforaphane Microcapsules via O/W Emulsion: Development, Characterization, and Application in Functional Yogurt

**DOI:** 10.3390/foods15122176

**Published:** 2026-06-16

**Authors:** Yipsy Arozarena, Víctor Zambrano, Rubén Bustos, Silvia Matiacevich, Claudia De Paula, Andrea Mahn

**Affiliations:** 1 Department of Chemical and Bioprocess Engineering, Engineering Faculty, University of Santiago of Chile, Avenida Libertador Bernardo O’Higgins 3363, Central Station, Santiago 9170019, Chile; victor.zambrano@usach.cl (V.Z.); ruben.bustos@usach.cl (R.B.); 2 Department of Food Science and Technology, Technological Faculty, University of Santiago of Chile, Obispo Umaña 050, Central Station, Santiago 9170019, Chile; silvia.matiacevich@usach.cl; 3 Department of Food Engineering, Engineering Faculty, University of Córdoba, Cra. 6 #77-305, Monteria 230002, Colombia; cdepaula@correo.unicordoba.edu.co

**Keywords:** sulforaphane, stabilization, gum arabic, dairy products, functional foods

## Abstract

Sulforaphane (SFN) is an anti-cancer isothiocyanate occurring in Brassicaceae. SFN is decomposed by heat, oxygen, and alkaline conditions. Microencapsulation is a way to improve its stability. This work presents the development of SFN microcapsules using an oil-in-water emulsion with gum arabic (GA) as the wall material for incorporation into yogurt. The process for obtaining SFN microcapsules was optimized using response surface methodology. The optimal microencapsulation conditions were 7 min of stirring, an SFN/GA mass ratio of 0.7, and a surfactant concentration of 7%, resulting in an entrapment efficiency of 90.0 ± 3.0%, the highest reported. The microcapsules had a regular spherical shape with 0.5–5.5 µm diameter and no cracks. Freezing temperature (−4 °C) preserved 100% of SFN in the microcapsule for 90 days. Yogurt added with SFN microcapsules maintained physical and microbiological quality. SFN retention in yogurt after 30 days was 57% when microencapsulated, showing a 14-fold reduction in the kinetic degradation constant compared with free SFN, confirming the efficacy of this method.

## 1. Introduction

Sulforaphane (SFN) is a compound derived from the hydrolysis of glucoraphanin, a glucosinolate found in vegetables of the Brassicaceae family. It has been extensively studied along with other isothiocyanates (ITCs) for its anticancer and antimicrobial effects. The antioxidant and anticancer effects of SFN have been demonstrated in various studies, including in vitro assays showing its ability to induce apoptosis and cell cycle arrest, as well as in vivo models where SFN reduced tumor growth and oxidative stress [1,2]. SFN shows strong therapeutic potential not only in neurodegenerative diseases but also in cancer prevention and treatment through diverse mechanisms, including antioxidant effects, immune modulation, and modulation of cell signaling pathways [2]. Several studies have demonstrated that SFN reduces or inhibits cell proliferation in various types of cancer, including oral cancer [3], colon cancer [4], breast cancer [5], and lung cancer [6].

Despite the health benefits of SFN, its applications in food and dietary supplements are limited by its susceptibility to decomposition under typical food processing conditions [7]. Several SFN stabilization strategies have been investigated, such as inclusion complexes with cyclodextrin [8] spray drying [9], iron oxide–gold core–shell nanoparticles [10], simple and complex coacervation [11], micelles [12], nanostructured lipid carriers [13], gold nanoparticles [14], electrospraying [15], broccoli membrane vesicles, selenium nanoparticles [16], nanoliposomes [17] and emulsion oil to water [18].

Previous studies demonstrated that SFN can be stabilized by encapsulation, using polymers such as gum arabic (GA) or gelatin as wall materials, achieving entrapment efficiency (EE) of 39.8 ± 1.5% and 29.4 ± 2.9% for SFN encapsulated in GA and GA/β-ciclodextrin complex, respectively, through the spray drying method, concluding that the use of high temperatures generates the degradation of SFN [9]. SFN EE ranging from 10 to 17% has been reported for systems stabilized by complex coacervation using gelatin/GA and gelatin/pectin, followed by drying in a vacuum oven [11]. The study by Franklin et al. [19] reports that the use of O/W emulsions in topical cream formulations effectively stabilizes SFN. This result is consistent with more recent findings by Tian et al. [20], who demonstrated that incorporating an oil phase to stabilize isothiocyanate increases their stability and bioavailability in an in vitro digestion study. In our previous studies [18], the use of O/W emulsions emerged as a viable strategy for SFN stabilization, achieving encapsulation efficiencies (EE) of up to 65% when GA was employed as the wall material and Vaseline as the oily phase [18].

Vaseline is neutral fat, colorless, and has no strong odor. Although it is not commonly used in food applications, it has been investigated in certain microencapsulation processes for bioactive compounds due to its high chemical stability, which avoids potential interference or reactions with the active principles during lab-scale experiments. For instance, an investigation used liquid Vaseline as the oil phase in W/O emulsions to encapsulate maqui leaf extracts, achieving high retention of the extract’s antioxidant capacity [21]. Vaseline used to produce food products must comply with its designation as a food additive E905b, which is a commercially available product specifically intended for oral consumption. In future experiments, common edible oils are used.

GA is a highly branched, complex polymer widely used as a wall material in the microencapsulation of lipophilic compounds. It is a non-toxic material, broadly recognized as safe for human consumption, with excellent film-forming properties that enable the formation of thin, stable coatings that adhere effectively to lipophilic cores. Its high-water solubility facilitates easy incorporation into the continuous phase of emulsions. It is classified as a dietary fiber or a non-digestible carbohydrate, as it can pass through the small intestine without degradation or causing adverse effects on the digestive system [22].

Currently, there are no reports on the effects of incorporating microencapsulated SFN in food. Accordingly, SFN microcapsules were developed using an efficient method and evaluated for food application. Given that oil-containing food matrices can protect hydrophobic compounds and enhance emulsion stability, a dairy product was selected to validate the microcapsules as a functional ingredient. Among them, yogurt is attractive because functional ingredients can be added after fermentation and thermal processing. Therefore, the objective of this study was to develop SFN microcapsules for use as a functional ingredient in blackberry yogurt, to obtain a dairy functional food that delivers SFN as the active principle.

## 2. Materials and Methods

### 2.1. Raw Material

All procedures were carried out using deionized water. Broccoli seeds were sourced from a local distributor (Santiago, Chile). The following materials were used: SFN standard (Sigma-Aldrich, Schnelldorf, Germany); gum arabic (Sigma-Aldrich, St. Louis, MO, USA); oral Vaseline (Difem Pharma S.A., Santiago, Chile); Tween-80 (Merck, Darmstadt, Germany); Dichloromethane (Sigma-Aldrich, St Louis, MO, USA); acetonitrile (Merck, Darmstadt, Germany); ethanol (Merck, Darmstadt, Germany); sodium sulfate (Winkler, Santiago, Chile); NaOH (Merck, Darmstadt, Germany); *Lactobacillus de brueckii* ssp. *bulgaricus*, *Streptococcus thermophilus*, and *Bifidobacterium* (CAL Group SAS, Bogotá, Colombia); Potato Dextrose Agar (Sigma-Aldrich, St. Louis, MO, USA); Chromocult Agar (Merck, Darmstadt, Germany); Violet-Red Bile Glucose Agar (Sigma-Aldrich, Darmstadt, Germany). Raw milk was obtained from local milking (Montería, Colombia).

### 2.2. Experimental Design

Preliminary tests were conducted to determine the optimal composition of the microencapsulation system, focusing on emulsion stability, including the oily phase, system temperature, agitation time, and SFN/GA ratio (µg SFN/mg GA). Two factorial designs, 2^3^ and 2^2^, were conducted, with EE as the response variable.

The O/W emulsion method was optimized using response surface methodology (RSM) [23]. Statgraphics Centurion Version XV was used to create experimental designs and perform statistical analysis. The system conditions were evaluated using a Box–Behnken design with three replicates at the central point, and the effects of the independent variables were analyzed both individually and through their interactions. The independent variables were homogenization time (7, 11, and 15 min), SFN/GA mass ratio (0.7, 1.4, and 2.1 µg SFN/mg GA), and surfactant concentration (0.3, 0.5, and 0.7 g Tween-80/g emulsion). The factor levels were chosen based on preliminary microencapsulation experiments. The response variable EE, defined in Equation (1), refers to the proportion of SFN retained within the microcapsules compared to the initial amount of SFN used in the formulation.

(1) $$EE=\frac{amount\;of\;microencapsulated\;SFN}{amount\;of\;SFN\;used\;for\;emulsion}$$

where EE is entrapment efficiency.

### 2.3. SFN Microencapsulation

#### 2.3.1. Sulforaphane Obtention

SFN extraction was conducted following the methodologies of Campas-Baypoli et al. [24] and Zambrano et al. [18], with minor modifications. For optimal formation of SFN from glucoraphanin, broccoli seeds (15 g) were milled and dispersed in 24 mL of deionized water and incubated at 45 ± 1 °C in the RE300 water bath (Stuart, Staffordshire, UK). Upon completion of the 3 h incubation, the mixture was supplemented with 13 g of anhydrous sodium sulfate and subjected to two successive extractions with 100 mL of dichloromethane in a sonication bath (Sonics & Materials, Inc., Model VCX750/VCX500, Newtown, CT, USA) for 30 min each time. The organic phases were vacuum-filtered, pooled, and concentrated on a rotary evaporator (EV400H-V, Labtech RS.r.L., Sorisole, Italy) at 30 °C until complete removal of the solvent. The final oily residue was dissolved in 99.6% analytical-grade ethanol.

#### 2.3.2. Preparation of SFN Microcapsules

Microcapsules were prepared according to [18] with adjustments, using an O/W emulsion with GA as the wall material, Vaseline as the oil phase, and Tween-80 as a surfactant. The way of adding the components was modified using two steps: (1) mixing Vaseline and surfactant in a 1:1 (*w*/*w*) ratio where Tween-80 represents 3, 5 or 7% of total emulsion, followed by adding SFN extract at 11, 20 or 28% of total emulsion (µgSFN/g emulsion), with stirring for 1 min, and (2) adding 20% GA solution (*w*/*w*) in volumes of 6, 7 or 8 mL for a total emulsion volume of 10 mL, as stipulated by the experimental design. These steps and phase constituents represent a modification of our previous work [18], which considered the aqueous phase as a mixture of GA and surfactant (Tween-80) and the oil phase as a mixture of Vaseline and the SFN extract.

The resulting mixture was homogenized using a blade homogenizer (Ultra-turra TR 50, IKA-Werke GmbH, Staufen, Germany) operated at 8000 rpm and a controlled temperature of 20 °C in a water bath to favor interactions between the components for 7, 11, or 15 min. This procedure differs from the previous method, which lacked temperature control [18]. The emulsions were dehydrated in a vacuum oven (Cole & Palmer, model 60061 Holdpack, Vernon Hills, IL, USA) at 37 °C and 0.6 MPa for less than 24 h. The resulting microcapsules were ground in a porcelain mortar. The EE was determined as described in Equation (1).

### 2.4. Properties of SFN Microcapsules

Under the chosen conditions, the resulting microcapsules were evaluated for SFN content, size, microstructural features, and molecular-mixture composition.

#### 2.4.1. SFN Quantification

SFN quantification was performed by reverse phase HPLC, as described by Liang et al. [25] with minor modifications using a Shimadzu HPLC-DAD (Tokyo, Japan). One mL of SFN-rich extract was vacuum-filtered and subjected to solvent elimination in a rotary evaporator at 30 °C. Two mL of acetonitrile were used to dissolve the resulting residue and were passed through a 0.22 μm membrane filter before HPLC analysis. The column was a reverse-phase C18 (5 μm, particle size 250 4.6 mm) (Agilent Technologies, Santa Clara, CA, USA). The mobile phase consisted of 20% acetonitrile in water, which was linearly increased to 60% acetonitrile over 10 min, followed by a hold at 100% acetonitrile for 5 min to purge the column. The column oven was maintained at 30 °C, with a flow rate of 1 mL/min. Aliquots of 20 μL were injected, and SFN was detected at 254 nm. Quantification was achieved by comparison with a calibration curve prepared from an SFN standard (Sigma-Aldrich, Schnelldorf, Germany), dissolved in acetonitrile and injected at eight different volumes. Peak areas were correlated with the standard mass range (0.056–6.75 g), yielding a determination coefficient of R^2^ = 0.99 for curve linearity. Each analysis was performed in triplicate.

Extraction of SFN from the microcapsules was performed by weighing 500 mg of the dry microcapsules, adding 10 mL of dichloromethane, sonicating for 15 min, and allowing the mixture to stand for 1 h. The mixture was filtered through Whatman No. 41 paper, and the solvent was removed by rotary evaporation at 30 ± 2 °C and reconstituted in 2 mL of HPLC-grade acetonitrile before HPLC analysis.

#### 2.4.2. Optical Microscopy

The SFN microencapsulated emulsion and the dehydrated SFN microcapsules were observed using a optical microscope (Euromex Scope series 100–400 V, Arnhem, The Netherlands) equipped with a 40× objective. Their structure was visualized using Euromex ImageFocusAlpha software, version 1.3.7.27993. 20250313. The emulsion was observed directly, whereas the dried microcapsules were dissolved in Milli-Q water and placed on a microscope slide.

#### 2.4.3. Scanning Electron Microscopy (SEM)

The SFN microcapsules were washed with ethanol to remove excess Vaseline and obtain an oil-free powder; they were air-dried for 3 h. The samples were coated with a thin gold layer. SEM analysis was conducted on a TESCAN model Vega 3 Scanning Electron Microscope (Brno, Czech Republic), and images were acquired at magnifications of 250×, 2.00k×, and 10.00k×. The weight percentage was analyzed using the Bruker probe model Quantax, series 400a Sprite 1.9 software. All samples were fixed on a carbon conductive tape and coated with a thin layer of gold in a metallizer before being scanned at 250×–10.0k× magnification.

#### 2.4.4. Fourier Transform Infrared Spectroscopy (FTIR)

To characterize the microcapsules in terms of the presence and interactions of SFN with other components, Fourier Transform Infrared (FTIR) analysis using a Spectrum 2 equipment (Perkin Elmer, Llantrisant, UK), equipped with a Universal Attenuated Reflection Unit (ATR), was performed according to Soni et al. [13], with minor updates. For each spectrum, in absorbance mode, 16 scans from 4000 cm^−1^ to 300 cm^−1^ at a resolution of 4 cm^−1^ were accumulated and averaged. Spectral analysis was performed using Spectrum Software v. 10.04.02. The microcapsules obtained under optimal conditions, and each component of the emulsion system, were analyzed separately. The components of the emulsion system (SFN extract in ethanol, GA in solution, Tween-80, and Vaseline) were all in a liquid state, and the dried microcapsules were dissolved in distilled water.

### 2.5. Stability of Microencapsulated SFN Powder During Storage

To apply microencapsulated SFN in food products, it is essential to study the SFN stability and functionality during storage. In this context, a comparative stability study was conducted between microencapsulated SFN in dry powder form and free SFN dissolved in ethanol to evaluate their behavior under different storage temperatures. The stability of the dry microcapsules was evaluated following the methodology reported by Y. Wu et al. [9], with minor modifications. A total of 500 mg of powdered microcapsules were stored in 10 mL glass vials and subjected to various temperatures (−4, 4, and 25 °C) for 210 days, measuring the SFN content in the microcapsules every 30 days. The study at 70 °C was conducted for 24 h to simulate extreme thermal stress and predict long-term behavior. All samples were analyzed in triplicate, and a control was established as the SFN solution in ethanol without encapsulation. The retention fraction was calculated according to Equation (2).

(2) $$\%\;retention=\frac{Sulforaphane\;content\;in\;yogurt\;at\;time\;t}{Initial\;sulforaphane\;content\;in\;yogurt}$$

where t is the time.

### 2.6. Formulation and Characterization of Yogurt

#### 2.6.1. Yogurt Preparation

The production of blackberry yogurt was carried out at the Pilot Dairy Plant of the Faculty of Engineering of the University of Córdoba, Colombia. The elaboration followed the methodology proposed by Santillán-Urquiza et al. [26] with minor improvements. The whole milk was pasteurized at 85 °C for 30 min with constant agitation, then cooled to 42 °C. The milk was inoculated with a freeze-dried starter culture containing *Lactobacillus de brueckii* ssp. *bulgaricus*, *Streptococcus thermophilus*, and *Bifidobacterium* (CAL Group SAS, Bogotá, Colombia). The milk was incubated for 4 h at 42 °C until it reached an acidity of 70 ± 2° thorned. Subsequently, the samples were cooled to 4 ± 1 °C, after which SFN microcapsules (2% *w*/*w*) and blackberry flavoring were incorporated. Finally, the samples were shaken, manually packed into 2 L containers, and stored at 4 ± 1 °C.

Three samples were analyzed: modified yogurt with ethanol-washed microcapsules (YM1), modified yogurt with unwashed microcapsules (YM2), and yogurt with free SFN (YM3). In addition, a plain yogurt sample without SFN was included as a control, with parameters maintained under identical experimental conditions, thereby validating the comparison among the tested formulations.

#### 2.6.2. Physicochemical Properties of Yogurt

The yogurt’s physicochemical properties were analyzed after 24 h of processing. pH was measured using a digital pH meter (Hanna Instruments, Woonsocket, RI, USA) (AOAC 945.27) (AOAC, 2016). Titratable acidity (as % lactic acid) was determined by the titration method (AOAC 947.05) (AOAC, 2016) using 0.1 M NaOH. The fat content was measured using the Geber method (AOAC 200.18) (AOAC, 2016), and total solids were determined by weight difference, after drying in an oven at 70 °C (AOAC 990.16) (AOAC, 2016) for 24 h [27].

#### 2.6.3. Microbiological Count

For the microbiological analysis, 10 g of yogurt samples (YM1, YM2, and YM3) were mixed with 90 mL of buffered peptone water in a sterile stomacher bag and homogenized. The microbiological analyses followed Vanderzant [28]. Mold and yeast counts were performed by plating the sample on Potato Dextrose Agar (Sigma-Aldrich, St. Louis, MO, USA) plates and incubating at 37 °C for 24 h. The growth of E. coli and total coliforms was measured using Chromocult Agar (Merck, Darmstadt, Germany) and incubating at 37 °C for 24 h. The growth of enterobacteria was analyzed using Violet-Red Bile Glucose Agar (Sigma-Aldrich, Darmstadt, Germany), which was incubated at 37 °C for 72 h. Decimal dilutions and plate counting methods were used, with results expressed as CFU/mL. Bacterial counts were performed in triplicate.

#### 2.6.4. Stability of SFN in Yogurt During Storage

To evaluate SFN retention, a 40-day stability study was conducted under refrigerated storage (4 °C). Yogurt containing SFN microcapsules and yogurt with free SFN were evaluated. For each sample, 2 g of yogurt was taken, and 0.5 g of SFN microcapsules were added; a control sample was prepared using the same proportion of yogurt with SFN extract.

SFN quantification in 2 g of stored yogurt was performed by extraction with 10 mL dichloromethane. The mixture was sonicated for 10 min at 4 °C, then centrifuged at 10,000 rpm for 10 min at 4 °C to separate the phases. The supernatant was collected and dried under reduced pressure on a rotary evaporator, and SFN content was measured through HPLC. All samples were analyzed in triplicate. The SFN retention corresponds to the total content of the mixture because the microcapsules were broken during extraction. The retention fraction was calculated according to Equation (2).

#### 2.6.5. Sensory Evaluation

Sensory analyses were performed in individual sensory cabins located at the Sensory Analysis Laboratory of the Engineering Faculty of the University of Córdoba (Monteria, Colombia). Blackberry yogurt containing SFN microcapsules was subjected to an acceptance test conducted at the Sensory Evaluation Laboratory of the University of Córdoba, Colombia, following the methodology described by De Paula et al. [29] with slight modifications. The samples were evaluated for acceptability by 102 untrained potential consumers recruited verbally, before participation, and all subjects provided informed consent. A mixed nine-point hedonic scale was used, where 1 means “I dislike it extremely” and 9 means “I like it extremely”, evaluating the attributes of color, smell, aroma, flavor, texture, and global impression. In addition, purchase intent was determined on a five-point scale, where 1 means “I would not buy it” and 5 means “I would buy it.” The samples were coded with three-digit numbers and presented randomly in 20 mL plastic cups. The acceptability index (AI) was determined for each attribute according to Equation (3),

(3) $$AI\left(\%\right)=\frac{A*100}{B}$$

where A is the average grade obtained for the product and B is the maximum grade given to the product. The study was conducted in accordance with the Declaration of Helsinki and was approved by the Central Bioethics Committee of the University of Córdoba, Colombia (no protocol code was issued under the Committee’s regulations; date of approval: 15 September 2023).

### 2.7. Statistical Analysis

The emulsions prepared in this study were produced in triplicate, as were all analytical determinations. Parametric data obtained from the microcapsule analysis are presented as mean ± standard deviation. Data related to the preparation of microcapsules were evaluated using Statgraphics Centurion Version XV software through analysis of variance (ANOVA). Similarities and differences among samples in the physicochemical properties of yogurt and the results obtained for the acceptance and purchase intention test were analyzed using the analysis of variance (ANOVA) (*p* ≤ 0.05).

## 3. Results

### 3.1. Effect of Process Conditions on Entrapment Efficiency

To demonstrate the feasibility of the microencapsulation process, preliminary trials were conducted, and the corresponding data are provided in Tables S1 and S2. The first experimental step involved evaluating the effect of temperature and the incorporation of the oil phase on the EE of SFN as a preliminary analysis (Table S1). The effects of the oily phase and temperature on the EE were analyzed, and no statistically significant differences were found for either parameter. The EE values for samples with Vaseline tend to be slightly higher than those without Vaseline, reaching a maximum of 42%.

The previous study reported lower values than those obtained in this work, in which neither surfactants nor oily material were used, with 12.2 ± 0.1% SFN EE in microcapsules obtained by vacuum oven drying via the complex coacervation method with gelatin/GA as the wall material and SFN ethanolic extract [11]. Another study reported an EE of 39.8 ± 1.5% when using GA as the wall material and applying spray drying. This method requires high processing temperatures and does not incorporate an oil phase, which may partly explain the lower EE values obtained [9]. However, the EE obtained is lower than that reported in a previous study [18] where the EE had an optimal value of 65 ± 4% and a surfactant to oil ratio (SOR) equal to 1 was used; in the preliminary study, it was 1.3. From this, we can deduce that the stability of the O/W emulsion with Vaseline as oil phase improves with a SOR value of 1, so it was used in the next step.

At 20 and 40 °C, no statistically significant differences were observed according to analysis of variance (ANOVA); therefore, 20 °C was selected as the reference temperature, given that SFN begins to degrade above 40 °C [30]. Moreover, 20 °C yielded the highest EE value.

The 2^3^ factorial design (Table S2) was used to evaluate the system behavior with and without Vaseline as the oil phase, at different agitation times and SFN/GA ratio, while maintaining a constant SOR value of 1. This design was expanded after the initial 2^2^ trials, where temperature was found to be non-significant and could not be further modified due to experimental constraints, thus excluding it from the optimization plan. The highest EE was 76% (SOR = 1, SFN/GA ratio = 0.7, and agitation time of 15 min). In our previous study, the EE was lower than this design, in emulsions with Vaseline as the oil phase using ice in homogenization, SOR = 1, surfactant concentration 8%, and SFN/GA ratio of 1 [18]. A controlled temperature of 20 °C promotes greater interaction between Tween-80 and SFN and GA, and it also favors the solubilization of SFN in the oily phase, thereby preventing its decomposition during drying.

### 3.2. Optimization of the SFN Microencapsulation Process

The microencapsulation conditions that maximize SFN EE were identified using a Box–Behnken design (see Table 1). The parameters selected for the experimental design were SFN/GA ratio, stirring time, and Tween-80 concentration (CT80); the response variable was EE. The highest EE was obtained in run 9 (90.0%) using the lowest SFN/GA ratio and the highest Tween-80 concentration. The lowest EE was in runs 11 and 12 (46.0 and 46.7%, respectively), using the highest SFN/GA ratio and CT80.

**Figure 1:**
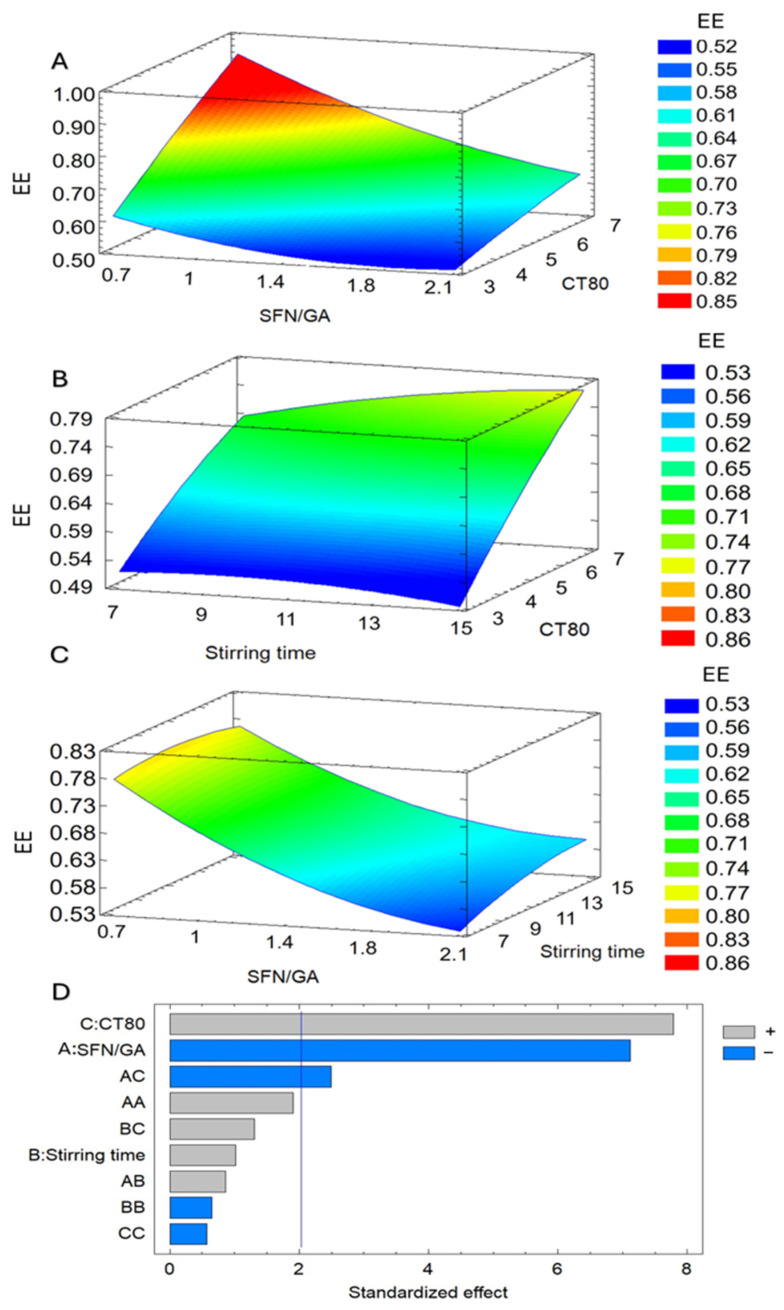
Optimization of design parameters and their effects on the SFN entrapment efficiency (EE) microencapsulation process. (**A**) SFN/GA vs. CT80 for stirring time: 11 min; (**B**) stirring time vs. CT80 for SFN/GA: 1.4; (**C**) SFN/GA vs. stirring time for CT80: 0.05, and (**D**) the Pareto plot.

The Pareto chart and response surface diagrams for SFN are presented in Figure 1. In the Pareto diagram in Figure 1D, the parameter CT80 had a significant positive effect on EE (*p*-value = 0.0000); low Tween-80 (3%) in the system resulted in an EE below 70.0%, while when CT80 is 7%, the EE increased. The SFN/GA had a significant adverse effect on EE (*p*-value = 0.0000); this suggests that the higher the SFN content in the system, the lower the retention of SFN in the microcapsule, indicating a limit on the amount of SFN content that can be loaded. The interaction between SFN/GA and CT80 significantly reduces EE (*p*-value = 0.0177). Therefore, higher EE was obtained with a lower SFN/GA ratio and higher CT80. Regarding stirring time, there is no statistically significant difference.

The highest EE (90.0 ± 4.1%) is close to that obtained when using lyophilization as a drying method for allyl isothiocyanate microcapsules obtained by complex coacervation with gelatin/GA, achieving an EE of 94.2 ± 2.3 [31]. Compared with our previous study [18], the way the components are mixed is crucial to emulsion stability. In general, the emulsifier is dispersed in the continuous phase before homogenization [32], as in our earlier method. However, according to reports in the literature, the mixture of surfactants in the dispersed phase as a first step generates greater stability than the addition in the continuous phase [33,34]. Another factor that determines stability is the emulsifier concentration. In our earlier research, the percentage was 8%; however, in the current work, this value decreased because excessive emulsifier concentration leads to flocculation and instability due to interface saturation. Furthermore, excessive emulsifier concentration causes flocculation and instability due to bridging or depletion attraction [35].

The regression model describing EE as a function of the experimental factors at coded levels is shown in Equation (4), with CT80 = 6.9%, an SFN/GA = 0.7, and a stirring time of 12.6 min. In Equation (4), EE is the entrapment efficiency, CT80 is the concentration of Tween-80, SFN/GA is the ratio between SFN and GA, and t is the stirring time. The regression model explained 79.2% of the variability in the response, indicating that it adequately represents the behavior of the system.

(4) $$\begin{array}{l} EE=0.64-0.10*\frac{SFN}{GA}+0.02*t+0.11*CT80+0.04*{\frac{SFN}{GA}}^{2}+0.02*\frac{SFN}{GA}*t-0.05*\frac{SFN}{GA}*CT80-0.01\\ \;\;\;\;\;\;*t^{2}+0.03*t*CT80-0.01*{CT80}^{2} \end{array}$$

The response surface plots in Figure 1A,B show that the response decreases as CT80 changes from high to low levels. The phenomenon of hydrophobic interactions in emulsions was described. This can cause the aggregation of oil droplets in aqueous phases (and vice versa), especially at low surfactant concentrations [36]. Stirring time has an optimum value within the ranges studied, located between 11 and 13 min, as shown in Figure 1B,C.

No significant effect of stirring time was observed on EE, so the shortest time (7 min) was used, as less work energy was required during this time. Under these conditions, the regression model predicts an EE of 94.8%. Experimental validation of the regression model’s optimal setting yielded an EE of 90.0 ± 3.0%, a result lower than that indicated by the regression model. This value agrees with the highest EE obtained in the experimental matrix. Accordingly, the model predicted the experimental response with a 3% deviation, consistent with the statistical design’s confidence level.

In Figure 1A,B, it can be observed that the highest EE coincides with one of the vertices of experimental space; therefore, it is not possible to ensure that it is indeed a maximum. To verify if it is a maximum, it would be necessary to run emulsion runs with higher CT80 and lower SFN/GA.

An extension of the optimization model with outward points (SFN/GA of 0.5, CT80 of 9%, and stirring time of 7 min) was implemented, yielding an EE of 57.3 ± 2.3%, a lower value than that obtained in the model. It agrees with the findings of Mujica et al. [37], which indicate that an excess of surfactant can translate into an increase in the polydispersity index. This parameter is closely related to the particle size, where high values of this index translate to emulsion instability and lower EE. This result corroborates the EE finding a maximum value within the experimental region.

The EE obtained under the optimal conditions identified in this work is higher than that reported by Soni et al. [14], who developed SFN-loaded nanostructured lipid carriers by emulsification and ultrasonication, achieving an optimized EE of 84.9 ± 3.8%, with the higher lipid content resulting in the best observed EE. The use of the organic phase increases the stabilization of SFN, considering the order of addition of the components to create better chemical bonds. As reported in our earlier findings, ice was used for homogenization, suggesting that a controlled temperature of 20 °C promotes stronger interactions between the bonds that stabilize Tween-80 with SFN and GA. Also, this favors the solubilization of SFN in the oil phase, in addition to positively affecting the previous solubilization of SFN with the oil medium and the surfactant.

Vacuum oven drying was selected for this study because the experiments were conducted at a laboratory scale, where smaller volumes are handled. Under these conditions, drying the emulsion in thin layers allowed greater material recovery, ensuring a sufficient sample for subsequent analysis. This approach provided a practical and efficient solution for laboratory conditions, while also preserving the properties of the microcapsules. In addition, this method is more cost-effective than lyophilization, as it requires lower vacuum levels and does not involve freezing, while still preserving the properties of the microcapsules.

### 3.3. Characterization of SFN Microcapsules

#### 3.3.1. Optical Microscope Observation

**Figure 2:**
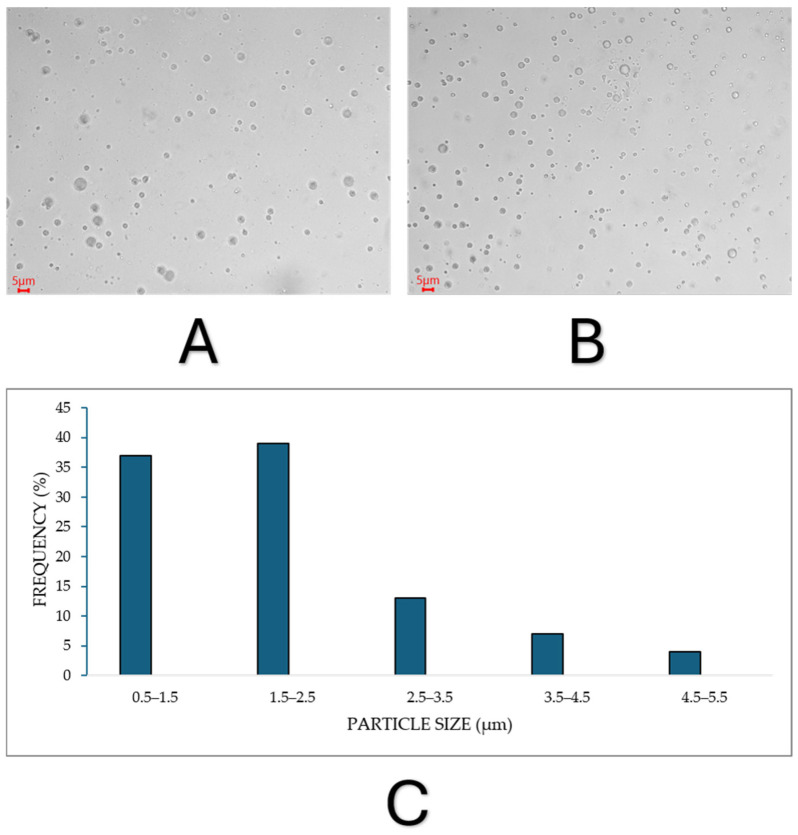
Optical microscope observation (40× objective) (**A**): resuspended dehydrated microcapsules; (**B**): microcapsules emulsion; and (**C**): particle size distribution plot.

The emulsion of microcapsules and the dehydrated microcapsules were observed under an optical microscope (Figure 2A). The particles in the emulsion were spherical and homogeneous in size and shape, forming multicores and droplets within droplets, as shown in Figure 2A,B. The dried microcapsules were resuspended in distilled water and showed uniformly spherical-shaped particles, well separated from one another and homogeneous in size (Figure 2B).

The highest particle size frequency ranged between 1.5 and 2.5 µm, as shown in Figure 2C. Emulsion stability is closely related to particle size; smaller particles confer greater stability. This result is consistent with the higher EE obtained in this study compared to our earlier work, which reported a particle size of 6.9 ± 2.3 µm.

**Figure 3:**
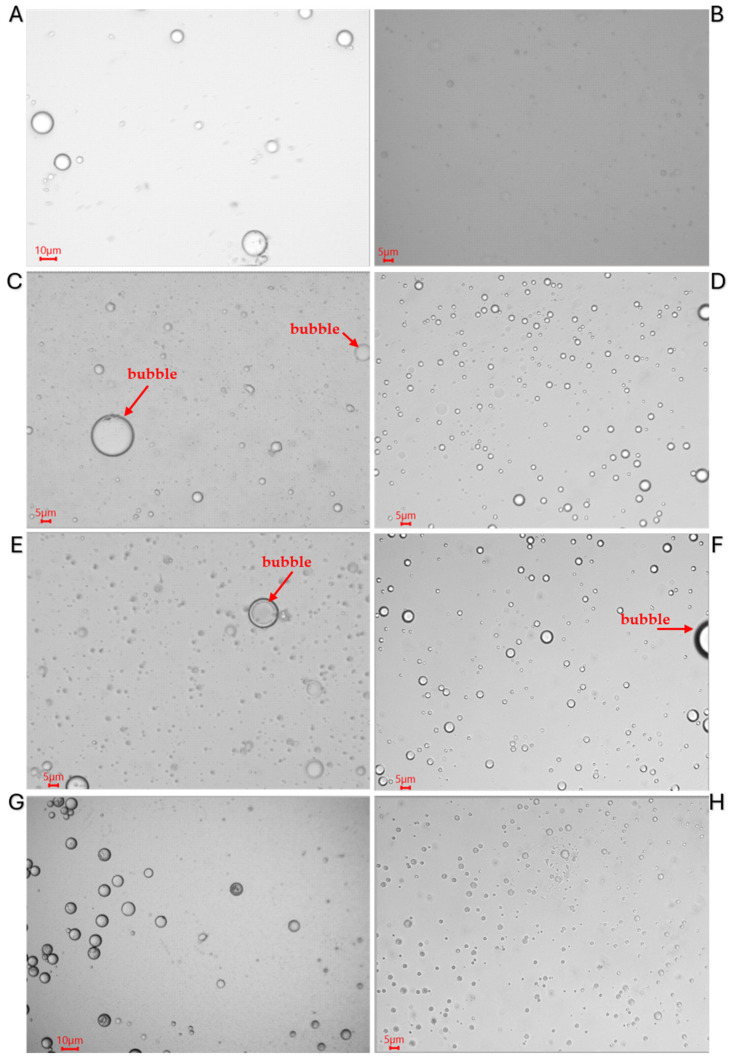
(**A**) SFN ethanol/water extract; (**B**) GA solution; (**C**) SFN ethanol/water extract and GA aqueous solution; (**D**) Tween-80 aqueous solution; (**E**) Tween-80 aqueous solution and SFN ethanol/water extract; (**F**) Vaseline aqueous solution; (**G**) Vaseline aqueous solution and SFN ethanol/water extract; (**H**) microcapsule from liquid emulsion.

The behavior of the particles of each component in the emulsion system was analyzed separately using optical microscopy. The SFN extract obtained from broccoli seed contains other compounds, primarily unsaturated fats, with a reported polyunsaturated fatty acid content of 71.39% [38]. It explains that there are fat droplets in Figure 3A with varying sizes, showing one inside the other. The SFN extract likely contains additional compounds besides SFN, including various oils and co-extractives, which may exert a protective effect [39].

Furthermore, in the mixture of SFN extract with Vaseline, the Vaseline droplets are less agglomerated (Figure 3G) than when the Vaseline is alone (Figure 3F). The ethanolic extracts of SFN contained significant amounts of phenolic compounds, which showed high antioxidant activity [40]. Then, the SFN extract could function as a surfactant in the mixture, thereby reducing the interfacial tension between the Vaseline droplets. Likewise, the oily component coats the SFN molecule, preventing its decomposition during preparation due to the presence of oxygen, light, and heat. This phenomenon is absent in complex coacervation, where no oily phase is used, which explains the low efficiencies obtained by [11].

#### 3.3.2. Morphological Characterization

**Figure 4:**
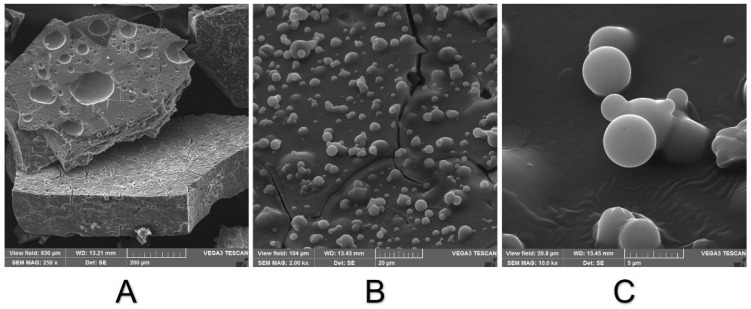
SEM micrographs of SFN powder microcapsules obtained by O/W emulsion with GA as wall material, ethanol washed and air dried, magnification 250× (**A**), 2.0k× (**B**), and 10.0k× (**C**).

The SFN microcapsules were visualized as homogeneous, smooth, and rounded, without noticeable cracks or deformations (Figure 4). These results support optical microscopy observations, showing round, intact, uniform particles. The particle size defined in this analysis ranged from 1.97 to 4.82 µm with an average of 2.6 ± 1.1 µm, agreeing with the size range determined by optical microscopy.

A study of SFN microencapsulation in different polymers, including maltodextrin, showed micrographs in which the microparticles exhibited more irregularities than in the current study, attributed to rapid water evaporation during spray drying [41]. Another feature the authors observed was agglomeration of larger particles (9.5 µm). Spray drying was employed for SFN microencapsulation, with maltodextrin selected as the wall matrix [9]. The resulting particles were rough and cracked, reducing the protective effect of SFN, in agreement with the low EE obtained (39.1 ± 2.6%).

#### 3.3.3. FTIR Profiles of SFN Microcapsules and Emulsion Components

**Figure 5:**
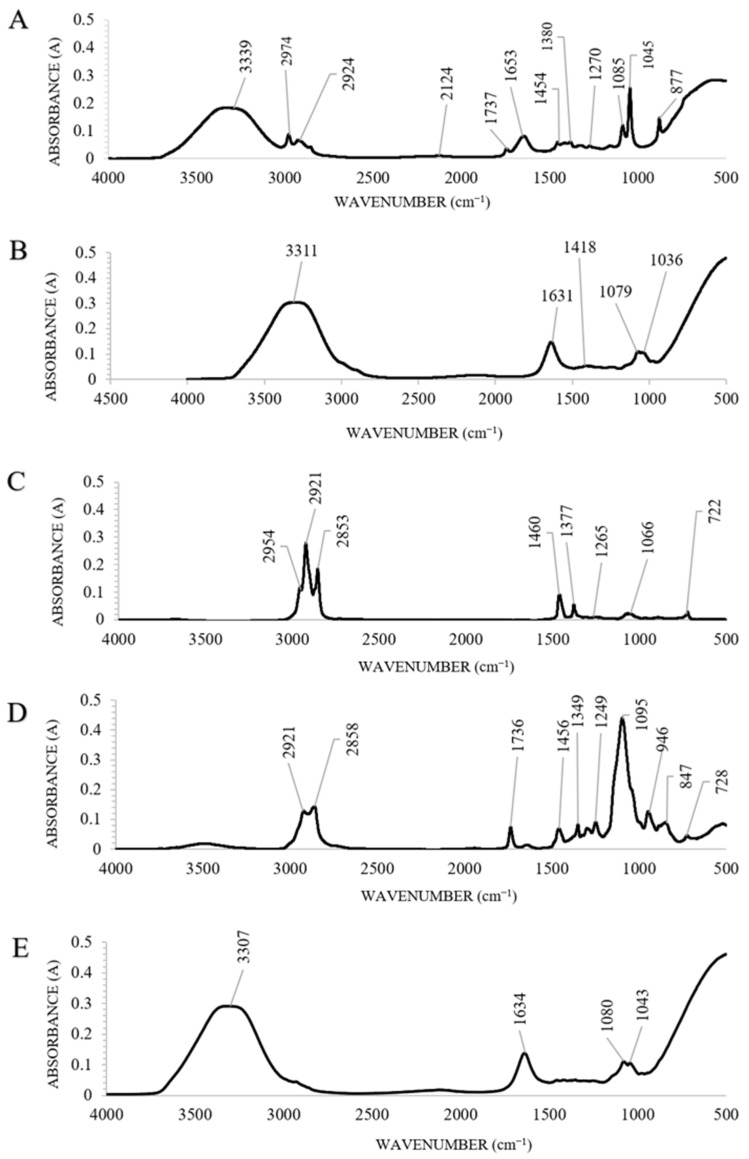
FTIR spectra of (**A**) SFN extract, (**B**) gum arabic, (**C**) Vaseline, (**D**) Tween-80, and (**E**) SFN microcapsules.

The FTIR spectra of both SFN microcapsules and the emulsion system components are displayed in Figure 5. Figure 5A shows typical SFN bands (1045 cm^−1^) belonging to the sulfoxide functional group (S=O), the bands 2124 cm^−1^ belonging to the isothiocyanate group (N=C=S), and the band 1737 cm^−1^ corresponds to the ester functional group (-COO) of triglycerides, which corroborates the presence of fat in the SFN extract.

In addition, the band 1454 cm^−1^ corresponds to the stretching vibration of the C-S bonds. Figure 5B shows characteristic bands for GA, 1631 cm^−1^ belonging to the carbonyl functional group (C=O). Carbohydrate-associated OH stretching bands at 3311 cm^−1^ and symmetric –COO^−^ stretching at 1418 cm^−1^ were also detected. Figure 5E shows the analysis of the microcapsules, the typical bands of each of the components disappear and that of SFN (band 1043 cm^−1^) appears with an absorbance below that analyzed in the extract, which indicates that there is a low amount of SFN on the surface, and a strong interaction between the components that keep the SFN associated and stabilized within the microcapsule, showing GA as the predominant component.

### 3.4. Storage Retention of Microencapsulated SFN

**Figure 6:**
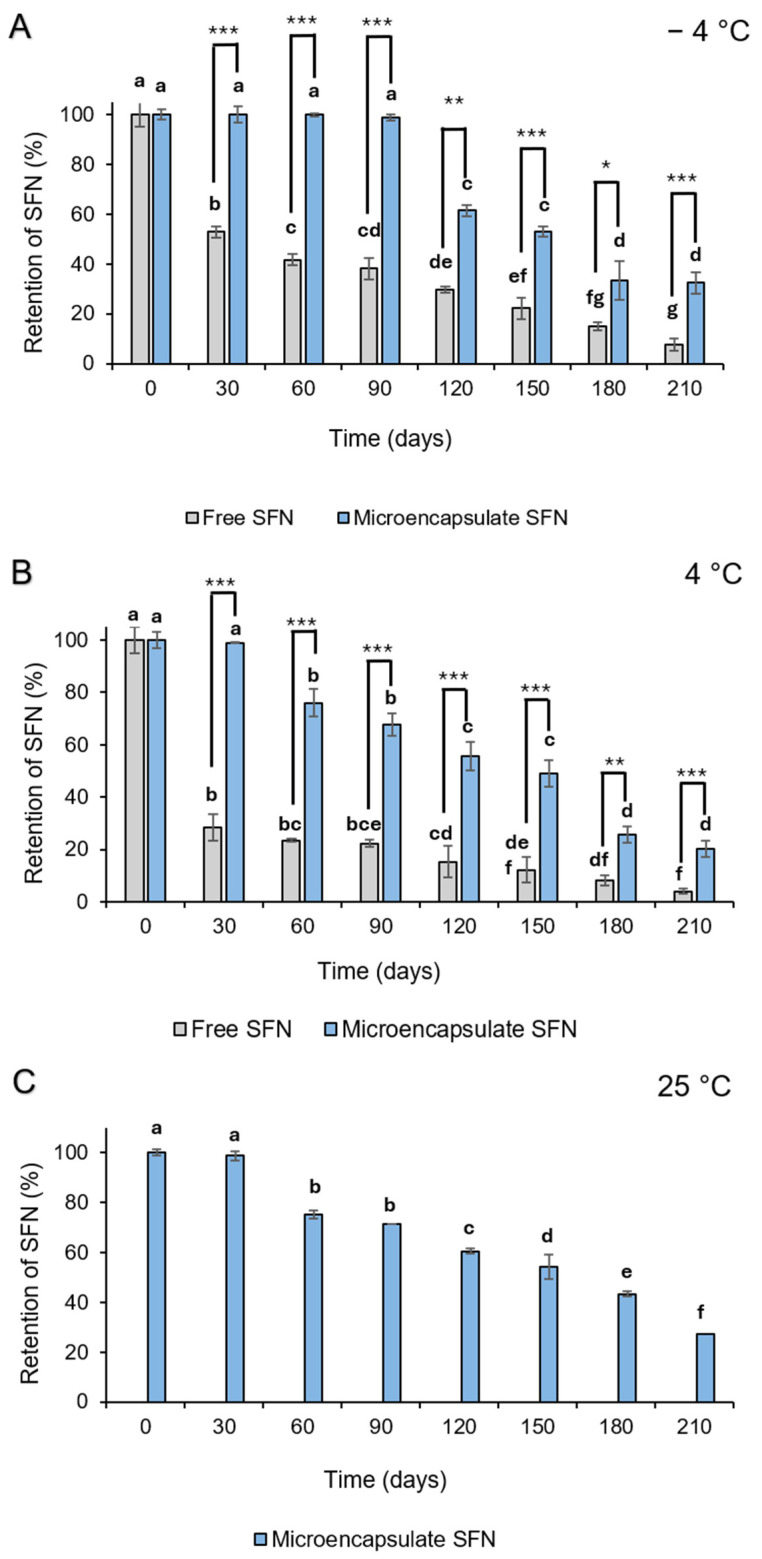
Retention of microencapsulated and free SFN during storage for 210 days at temperatures of −4 °C (**A**), 4 °C (**B**), and 25 °C (**C**). Differences between treatments (free SFN vs. microencapsulated SFN) are indicated by asterisks (* *p* < 0.05; ** *p* < 0.01; *** *p* < 0.001), while differences between storage times are shown by different letters for each treatment.

Figure 6 shows the retention of free and microencapsulated SFN during 210 days of storage at −4 °C (A), 4 °C (B), and 25 °C (C). In all tested conditions, microencapsulated SFN exhibited significantly higher retention values than free SFN, confirming its enhanced stability. Statistical differences between treatments were determined using one-way ANOVA, whereas significant differences among storage times within each treatment were identified by Tukey’s test (*p* < 0.05).

The highest retention of microencapsulated SFN was observed at freezing temperature (−4 °C), maintaining 100% concentration up to 90 days (Figure 6A), with no significant differences among storage times. This indicates minimal variability and effective protection by the encapsulation matrix.

Under refrigeration (4 °C), the retention of free SFN decreased to one-third of the value of microencapsulated SFN (Figure 6B). In contrast, microencapsulated SFN showed no significant differences between the initial time and 30 days of storage (0–30 days). From 90 days onwards, progressive differences were observed. This reflects a slower, but statistically detectable, degradation.

At room temperature (25 °C), free SFN was not detectable, whereas microencapsulated SFN remained stable up to 30 days and maintained retention above 50% up to 180 days (Figure 6C). This result suggests that the obtained powder can be incorporated into dry products under these conditions.

The statistical results support that the difference between treatments is significant and that the variation between days depends on the temperature: homogeneous in freezing, but progressive and marked in refrigeration.

These results demonstrate the ability of the microencapsulation process, under the optimized conditions, to stabilize SFN during prolonged storage. No comparative studies on the stability of SFN microcapsules under these conditions have been reported; therefore, the advantage of this study lies in identifying the appropriate storage conditions to ensure greater durability of the product.

**Figure 7:**
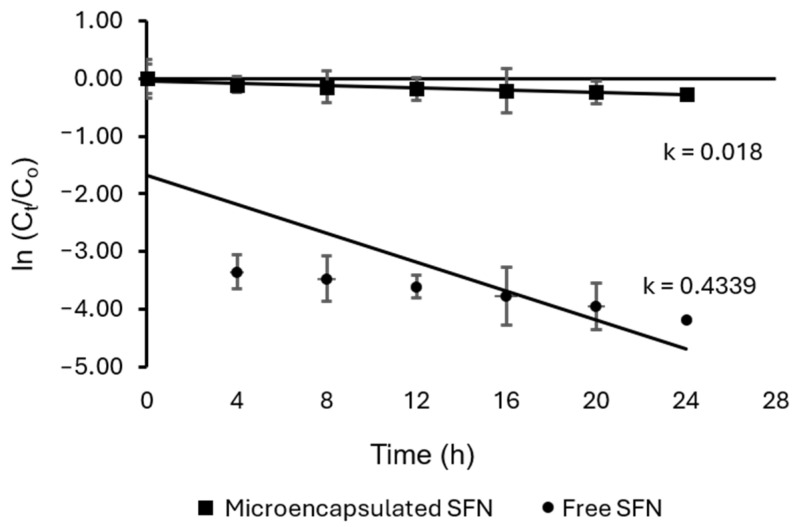
Plot of C_t_/C_0_ versus time (t) for SFN microencapsulated in powder form (■) (Eq. ln(C_t_/C_0_) = −0.0099t − 0.0415) and free SFN dissolved in ethanol (●) (Eq. ln(C_t_/C_0_) = −0.1255t − 1.6847) at 70 °C for 24 h. The equations were fitted to the first two experimental points to estimate the degradation rate constant (k) in the initial phase, during which free SFN shows a rapid decrease. Solid line represents the trend line.

In the accelerated degradation study, both free and microencapsulated SFN were subjected to 70 °C for 24 h. The results are presented in Figure 7. To describe the degradation behavior, a first-order kinetic model was applied, in which the degradation rate is proportional to the compound concentration. However, in the case of free SFN, a very rapid decrease in concentration was observed at the beginning of the experiment, so subsequent points did not adequately represent the initial kinetics. Thus, only the first two experimental points were considered to better estimate the initial degradation rate. The value of the rate constant (k) was lower for microencapsulated SFN compared with free SFN, evidencing the protective effect of microencapsulation against elevated temperatures.

In our previous study, the degradation rate constant was 0.0296 h^−1^ when microcapsules were subjected to 70 °C [18], whereas in this work, a value of k = 0.018 h^−1^ was obtained. This result contrasts with the higher encapsulation efficiency achieved, supported by scanning electron microscopy observations that confirmed the absence of cracks in the analyzed particles.

### 3.5. Incorporation of SFN Microcapsules in Stirred Yogurt

After the milk was collected, physicochemical characterization was performed, obtaining the results presented in Table 2. The results obtained for whole milk were within the recommended range for yogurt production, meeting the desirable parameters for good processing.

The yogurt was prepared, and SFN microcapsules were added after sweetening, due to the requirement for low temperatures and improved microcapsule preservation. Three conditions were studied: blackberry yogurt with microcapsules previously washed in ethanol (YM1), blackberry yogurt with unwashed microcapsules (YM2), and blackberry yogurt without microcapsules (YM3). For the study, a sample of washed microcapsules was employed to identify potential interferences within the food matrix. Observations performed by SEM showed that the washed microcapsules remained intact and without cracks, suggesting adequate protection of the active compound.

#### 3.5.1. Physicochemical Analysis

The physicochemical parameters were within the limits established by the Codex Alimentarius STAN 243-2 [42] (Table 2). There are significant differences between sample YM3 and samples YM1 and YM2 according to Tukey’s test. Therefore, it can be concluded that incorporating unwashed SFN microcapsules into yogurt increases the total fat content of the product, attributable to residual Vaseline on the microcapsules’ surface. This change in fat content, compared with the control, is associated with lower acidity in the product, although still within permitted limits. In contrast, the addition of washed microcapsules produces no significant differences compared with the control. Regarding the other parameters, the samples showed no significant differences, confirming that incorporating washed and unwashed microcapsules does not negatively influence these attributes.

Although no significant differences were observed between the samples, YM1 and YM2 showed slightly lower acidity than YM3, and the pH of YM1 and YM2 increased relative to the control, due to Vaseline falling within a basic pH range.

Unlike milk fat, which has a triglyceride structure composed of short- and medium-chain fatty acids, Vaseline is a mixture of high-molecular-weight saturated hydrocarbons of mineral origin. This structural difference has physicochemical implications; while milk fats can contribute to the release of free protons (H^+^) upon degradation, thereby increasing acidity, Vaseline does not easily undergo hydrolysis nor actively participate in reactions that release protons [43].

#### 3.5.2. Yogurt Microbiological Analysis

The microbiological analysis showed no growth for *E. coli*, total coliforms, *Enterobacteriaceae*, mold, and yeasts in dilutions 10^−4^ and 10^−5^. These results were expected under good manufacturing practices, using components considered GRAS (Generally Recognized as Safe). The yogurt containing SFN microcapsules was produced in accordance with Chilean Ministry of Health regulations for dairy products, which only consider Enterobacteriaceae, molds, and yeasts as analytical parameters. It is important to note that this is a novel product, not previously studied in food matrices, and therefore represents the first attempt at its incorporation into food applications. For more comprehensive studies and its eventual commercialization, further microbiological analyses will be necessary.

#### 3.5.3. Stability of Yogurt with Microcapsules in Refrigerated Storage

**Figure 8:**
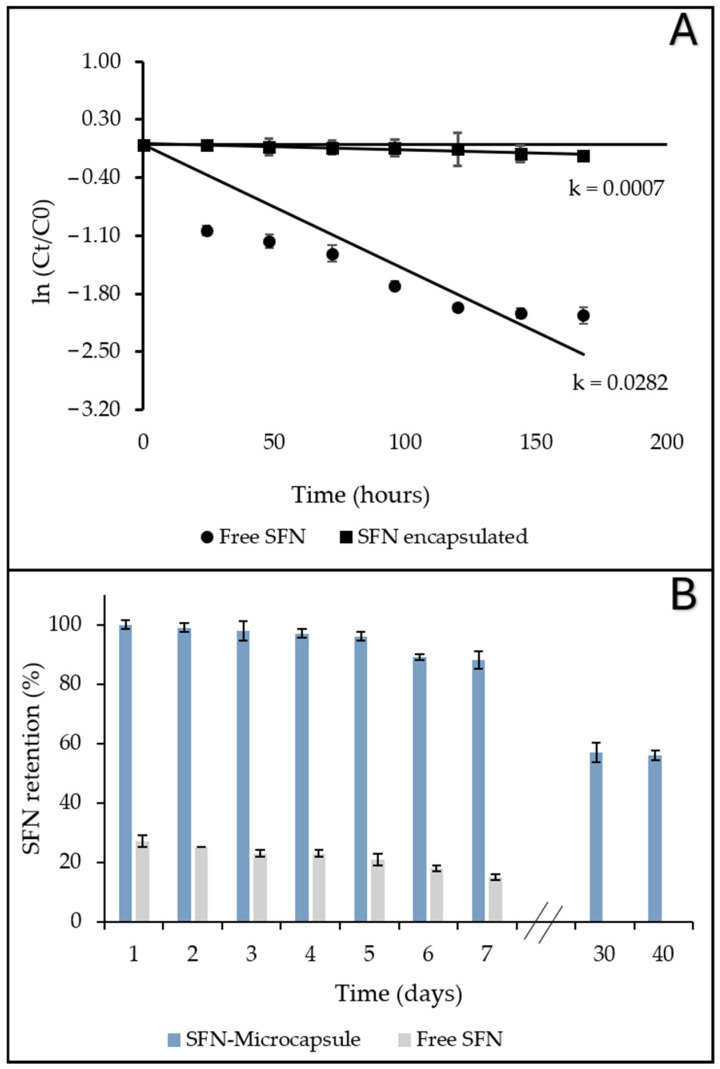
Stability of microencapsulated and free SFN in blackberry yogurt stored at 4 °C. (**A**): Degradation kinetics of microencapsulated (y = −0.0008x + 0.0129) and free SFN (y = −0.011x − 0.4874) in yogurt stored at 4 °C. The equations were fitted to the first two experimental points to estimate the degradation rate constant (k) in the initial phase, during which free SFN shows a rapid decrease. Solid line represents the trend line. (**B**): SFN retention in yogurt during 40 days of storage. Perpendicular lines delimit the 7-day kinetic study from the complementary 30–40-day stability study in yogurt. Free SFN was not detected at 30 and 40 days.

The yogurt was stored for 7 days at 4 °C to evaluate the stability of both microencapsulated and free SFN, through a kinetic study of compound degradation by calculating the degradation constant. In this case, microencapsulated SFN was incorporated into the yogurt using unwashed microcapsules, to employ them as obtained after the dehydration process. In contrast, free SFN was incorporated as an ethanolic extract, which may affect yogurt properties; therefore, physicochemical parameters were not monitored over time. The main objective was to demonstrate the degradation rate of SFN in the food matrix and to confirm the protection provided by microencapsulation. Yogurt belongs to the group of dairy products with a short shelf life, typically around 3 weeks or less [44]. Considering the above, the SFN content was measured to assess its retention in the food during refrigerated storage until expiration, and the SFN degradation constant was determined. Figure 8 shows the degradation constant of SFN in yogurt stored for 7 days and SFN retention after 30 days of storage.

Figure 8A shows the degradation kinetics of microencapsulated and free SFN in yogurt. Microencapsulated SFN showed a degradation kinetics constant (k) of 0.0008 [h^−1^], being 14-fold lower than that of free SFN (0.011 h^−1^). The coefficients of determination (R^2^) were above 86%, thus allowing an acceptable estimation of the constants. This result confirms that the microencapsulation method developed in this work effectively protects SFN from decomposing during storage at low temperatures.

Figure 8B shows a greater SFN loss in yogurt containing free SFN, with a 7-day loss of 70% and total compound loss after 30 days of storage. Microencapsulated SFN remained unchanged in yogurt for up to 5 days, decreasing to 88% retention on day seven. After 30 storage days, 57% of the initial SFN content was retained in the food.

Retention values at 30 days were obtained from a complementary stability study, where free SFN was not detected. The degradation constant was calculated from the 7-day kinetic follow-up; therefore, the slight deviation (~3.6%) between the expected and HPLC-observed values is attributed to model extrapolation and analytical variability.

These results demonstrate that microencapsulation is an effective strategy for stabilizing SFN and enabling its incorporation into dairy foods as a functional ingredient, maintaining its bioavailability throughout the product’s shelf life. In contrast, the addition of free SFN was performed as an ethanolic extract, which could alter yogurt properties and therefore limit its direct application in food products. For this reason, physicochemical parameters of yogurt with free SFN were not monitored over time. The main objective was to confirm that microencapsulation protects the active compound and allows its incorporation into the dairy matrix, overcoming the limitations associated with free SFN.

#### 3.5.4. Sensory Analysis

**Figure 9:**
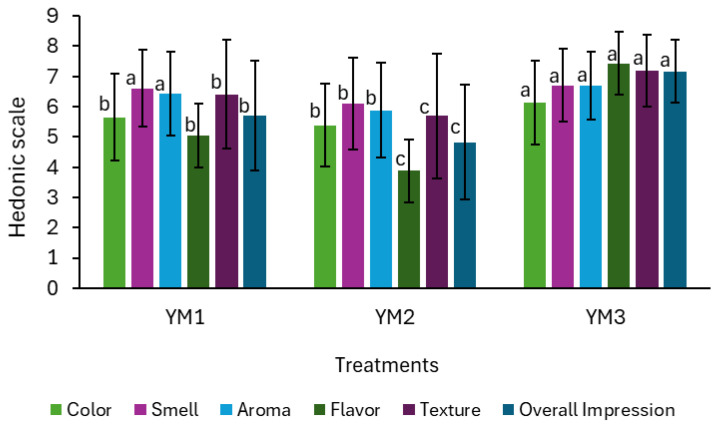
Graphical representation of attribute acceptance test by descriptive analysis using the nine-point hedonic scale. Differences between treatments are shown by different letters for each treatment: (YM1—modified yogurt ethanol-washed microcapsules); YM2—modified yogurt with unwashed microcapsules; YM3—modified yogurt without microcapsules.

Three samples were randomly presented to the panel. Figure 9 presents the results of the sensory attribute acceptance test and their corresponding scores. In the attribute acceptance test, there were differences between the formulations for all attributes (*p* ≤ 0.05) (see Table 3).

The color was located between the hedonic terms “I like it slightly” and “Indifferent” with no differences (*p* ≥ 5) for the YM2 and YM1 samples being different from the control, which had the highest score. The smell, aroma and texture were located between “I like it moderately” and “I like it slightly” with no differences for the YM3 and YM1 samples; the YM2 has the lowest score, which is related to the excess fat that remains on the surface of the microcapsules and provides a different smell compared to the control. The smell and aroma differ from each other in terms of how each one is perceived; the first is related to the perception of volatile substances through the nose and the second is the detection that originates after the food has come into contact in the mouth, the means of transmission being the mucosa of the palate [45].

The flavor was located between “I like it moderately”, “Indifferent” and “I dislike it slightly”, with the lowest score being the YM2 sample.

The overall impression remained between the terms “I like moderately”, “I like slightly” and “Indifferent” with the lowest score for the YM2 sample, which, despite the above attribute scores, did not indicate a general dislike for the sample. The YM1 sample was the most accepted among the treatments, which justifies the need to wash the microcapsules to remove excess fat from the product.

The purchase intent evaluated using a five-point hedonic scale revealed significant differences among treatments. Sample YM3 obtained the highest purchase intent score, corresponding to the category “Possibly I would buy it” (4.3 ± 0.9a), followed by YM1 with “Maybe I would buy it” (2.8 ± 1.3b), whereas YM2 showed the lowest acceptance, classified as “I probably would not buy it” (1.9 ± 1.1c). Statistical analysis (ANOVA followed by Tukey’s test, *p* < 0.05) confirmed that each treatment belongs to a different significance group, highlighting a greater willingness to purchase YM3.

The acceptability index value for color, smell, aroma, flavor, texture and the overall impression was determined for YM1 (67.40, 73.42, 71.35, 56.10, 71.24 and 63.40% respectively); YM2 (62.85, 67.76, 65.36, 43.13, 63.29 and 53.59% respectively) and YM3 (68.19, 655 74.51, 74.51, 82.57, 79.96, 79.63% respectively).

Regarding the value obtained for the AI of YM with the addition of microcapsules, according to de Paula et al. [29], a product with a percentage equal to or greater than 70% is considered acceptable to consumers. Based on the above, the control formulation was the only one that met these requirements. Formulation YM1 was the one that came closest to this value, with color, flavor, and overall impression below 70%.

While there is no established upper limit for SFN, studies in the literature have reported toxicity at doses of 150 to 300 mg/kg body weight [46]. FAO defines each country’s daily consumption of milk and dairy products. In Chile, the recommendation is one unit of yogurt per day for adults (175 g) [47]. The dose of SFN used in yogurt was 10.8 µg/g, it is not considered toxic at this concentration.

Yogurt with SFN microcapsules did not show high sensory acceptance, which is attributed to the lack of flavor enhancers. In yogurt production, it is common to add various additives, both natural and modified, to improve their physicochemical, textural, sensory, and rheological properties. Adding flavors to yogurt can enhance its sensory attributes and make it more appealing to consumers. Flavored yogurt may contain flavor enhancers, including other additives, such as acidity regulators, colorants, emulsifiers, packaging gases, stabilizers, and sweeteners [48]. Therefore, incorporating flavor enhancers is necessary in the formulation of yogurt with SFN microcapsules.

## 4. Conclusions

SFN microcapsules were successfully prepared using the O/W emulsion microencapsulation method with GA as the wall material. An improved method for manufacturing SFN microcapsules with the highest reported EE to date was established. The optimal conditions that maximized EE were SFN/GA: 0.7%, CT80: 7%, and stirring time: 7 min, achieving EE of 90.0 ± 3.0%, the highest value reported for this system. The microstructure of the capsules indicated rounded, smooth, and uncracked particles, with a size frequency between 0.5 and 5.5 µm. Freezing temperature (−4 °C) preserved 100% of SFN in the microcapsule for 90 days. The incorporation of SFN microcapsules into yogurt did not alter its physical properties but lowered the acceptability index, which improvements in yogurt formulation should address. A method for stabilizing SFN was developed that allows its use as a functional ingredient in foods, with microcapsules retaining approximately 57% of SFN after 30 days of storage, thereby demonstrating their protective effect and potential for extended shelf life.

## Figures and Tables

**Table 1:** Box–Behnken design of the independent experimental variables: agitation time, SFN/GA ratio, and Tween-80 concentration, and their effects on entrapment efficiency.

Samples	SFN/GA Ratio (μg SFN/mg GA)	Stirring Time (min)	Tween-80 Concentrations (g Tween-80/g Emulsion)	EE (%)
1	1.4	11	0.05	79.0 ± 1.0
2	0.7	7	0.05	79.0 ± 2.0
3	2.1	7	0.05	55.3 ± 2.9
4	0.7	15	0.07	75.3 ± 2.1
5	2.1	15	0.05	58.7 ± 4.0
6	0.7	11	0.03	65.7 ± 0.6
7	2.1	11	0.03	55.0 ± 4.0
8	1.4	11	0.05	57.3 ± 2.1
9	0.7	11	0.07	90.0 ± 4.1
10	2.1	11	0.07	58.7 ± 2.1
11	1.4	7	0.03	46.0 ± 1.7
12	1.4	15	0.03	46.7 ± 1.2
13	1.4	7	0.07	71.7 ± 4.0
14	1.4	15	0.07	83.0 ± 1.0
15	1.4	11	0.05	57.0 ± 3.0
Optimun	0.69	12.6	0.07	90.0 ± 3.0

**Table 2:** Physicochemical analysis of whole milk and yogurt.

Analysis	Milk	Milk Standard Conditions ^1^	YM1	YM2	YM3	Regulations of Fermented Milk ^2^
Acidity (%)	16.5 ± 0.1 (°Th)	16–17 (°Th)	1.2 ± 0.1 ^a^	1.2 ± 0.1 ^a^	1.3 ± 0.1 ^a^	min. 0.6
pH	6.5 ± 0.2	6.6–6.8	4.3 ± 0.1 ^a^	4.3 ± 0.1 ^a^	4.2 ± 0.1 ^a^	*
Total fat (%)	*	*	2.6 ± 0.1 ^a^	3 ± 0 ^b^	2.6 ± 0 ^a^	<15
Humidity (%)	*	*	78.0 ± 2.0 ^a^	78.0 ± 3.0 ^a^	79.0 ± 1.0 ^a^	*
Total solids (%)	*	*	22.0 ± 1.0 ^a^	22.0 ± 1.0 ^a^	21.0 ± 1.0 ^a^	min. 7
°Brix	9.0 ± 0.1	8–9	N/A	N/A	N/A	

Average of three replicates ± standard deviation. Different letters (a, b) in the same row indicate statistically significant differences (*p* ≤ 0.05). N/A (Not Applicable). ^1^ Data obtained from the Ministry of Health of Chile. ^2^ Values regulated according to Codex Alimentarius. * Data not reported.

**Table 3:** Attribute acceptance test and purchase intent of yogurt containing SFN microcapsules *.

Samples	Color	Smell	Aroma	Flavor	Texture	Overall Impression
YM1	5.7 ± 1.4 ^b^	6.6 ± 1.3 ^a^	6.4 ± 1.4 ^a^	5.1 ± 2.1 ^b^	6.4 ± 1.8 ^b^	5.7 ± 1.8 ^b^
YM2	5.4 ± 1.4 ^b^	6.1 ± 1.5 ^b^	5.9 ± 1.6 ^b^	3.9 ± 1.9 ^c^	5.7 ± 2.1 ^c^	4.8 ± 1.9 ^c^
YM3	6.1 ± 1.4 ^a^	6.7 ± 1.2 ^a^	6.7 ± 1.1 ^a^	7.4 ± 1.3 ^a^	7.2 ± 1.2 ^a^	7.2 ± 1.0 ^a^

* Different letters (a, b, c) in the same column indicate statistically significant differences (*p* ≤ 0.05) for the attribute based on a Tukey test. *n* = 102 consumer tasters. Values based on a nine-point hedonic scale (1 = “I dislike it extremely”, 9 = “I like it extremely”).

## Data Availability

The original contributions presented in this study are included in the article/Supplementary Materials. Further inquiries can be directed to the corresponding authors.

## Supplementary Materials

The following supporting information can be downloaded at: https://www.mdpi.com/article/10.3390/foods15122176/s1, Figure S1. Sulforaphane chromatogram in liquid extract from broccoli seeds; Figure S2: Sulforaphane chromatogram in microcapsules prepared under optimal conditions predicted by the experimental design; Table S1. Preliminary microencapsulation tests for different temperatures and oil phases by the O/W emulsion method. Factorial designs 22. Volume: 10 mL; Stirring time: 15 min; SFN/GA ratio (μg/mg): 1.2; Stirring speed: 8000 rpm; Wall material: GA; Surfactant: Tween 80 (8%) and SOR1 (mg Tween80™/mg Vaseline): 1.3; Table S2: Preliminary SFN microencapsulation tests for different stirred times, SFN/GA ratio (μg/mg) and oil phases. Factorial designs 2^3^. Volume: 10 mL; Stirring speed: 8000 rpm; Wall material: GA; Surfactant: Tween 80 (6%); Temperature: 20 °C, SOR1: 1.0; Oil phase: Vaseline; Table S3: Analysis of Variance for entrapment efficiency.

## Abbreviations

SFN	Sulforaphane
GA	Gum Arabic
GRAS	Generally Recognized as Safe
Tween-80	Polysorbate 80
CT80	Tween-80 concentration
SFN/GA	The relation between sulforaphane and gum arabic
EE	Entrapment efficiency
