# Supplementary material for: Sulforaphane Microcapsules via O/W Emulsion: Development, Characterization, and Application in Functional Yogurt
Source: Foods. 2026 Jun 16;15(12):2176. doi: 10.3390/foods15122176 (PMC13298518; doi:10.3390/foods15122176)
Supplement: Supplementary file 1 [file foods-15-02176-s001.zip › Supplementary Figures.pdf]

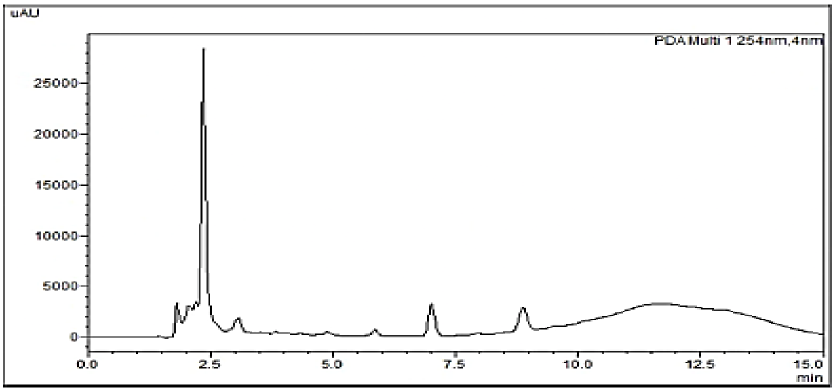

Figure S1. Sulforaphane chromatogram in liquid extract from broccoli seeds.

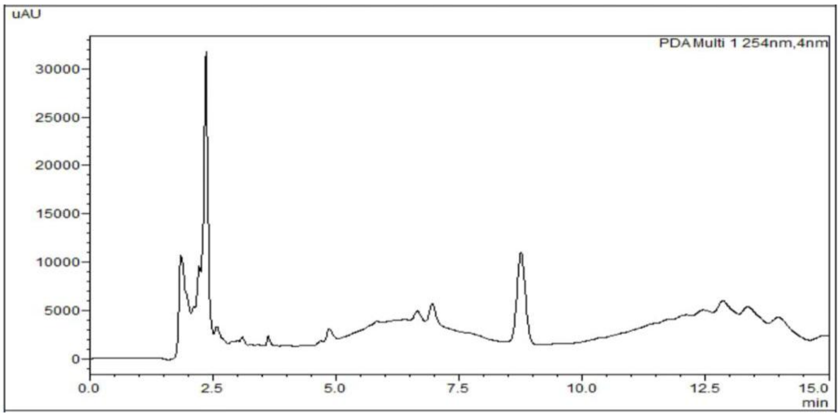

Figure S2: Sulforaphane chromatogram in microcapsules prepared under optimal conditions predicted by the experimental design.
