# Supplementary material for: Sulforaphane Microcapsules via O/W Emulsion: Development, Characterization, and Application in Functional Yogurt
Source: Foods. 2026 Jun 16;15(12):2176. doi: 10.3390/foods15122176 (PMC13298518; doi:10.3390/foods15122176)
Supplement: Supplementary file 1 [file foods-15-02176-s001.zip › Supplementary Tables.pdf]

Table S1. Preliminary microencapsulation tests for different temperatures and oil phases by the O/W emulsion method. Factorial designs 2^2. Volume: 10 mL; Stirring time: 15 min; SFN/GA ratio (µg/mg): 1.2; Stirring speed: 8000 rpm; Wall material: GA; Surfactant: Tween 80 (8%) and SOR[1] (mg Tween80™/mg Vaseline): 1.3.

Runs	Temperature (°C)	Oil phase	EE[2] (%)
1	20	0	30.2 ± 1.5
2	40	0	20.9 ± 2.2
3	20	Vaseline	42.0 ± 8.1
4	40	Vaseline	37.7 ± 1.1

[1]Surfactant-Oil Ratio
[2] Entrapment Efficiency

Table S2. Preliminary SFN microencapsulation tests for different stirred times, SFN/GA ratio (µg/mg) and oil phases. Factorial designs 2^3. Volume: 10 mL; Stirring speed: 8000 rpm; Wall material: GA; Surfactant: Tween 80 (6%); Temperature: 20°C, SOR[1]: 1.0; Oil phase: Vaseline.

Runs	Stirring time (min)	SFN/GA ratio (µg/mg)	Oily phase	EE[2] (%)
1	7	0.7	0	61.5 ± 8.6
2	15	0.7	0	66.0 ± 6.4
3	7	2.1	0	57.5 ± 6.9
4	15	2.1	0	61.3 ± 3.3
5	7	0.7	Vaseline	62.0 ± 4.8
6	15	0.7	Vaseline	76.0 ± 6.2
7	7	2.1	Vaseline	70.3 ± 7.9
8	15	2.1	Vaseline	73.5 ± 5.1

[1]Surfactant-Oil Ratio
[2]Entrapment Efficiency

Tabla S3. Analysis of Variance for entrapment efficiency

Fuente	Suma de Cuadrados	Gl	Cuadrado Medio	Razón-F	Valor-P
A:Ratio SFN GA	0,25215	1	0,25215	50,72	0
B:Stirring time	0,00510417	1	0,00510417	1,03	0,3183
C:CT80	0,301504	1	0,301504	60,65	0
AA	0,0179701	1	0,0179701	3,61	0,066
AB	0,003675	1	0,003675	0,74	0,3961
AC	0,0310083	1	0,0310083	6,24	0,0177
BB	0,00213675	1	0,00213675	0,43	0,5166
BC	0,00853333	1	0,00853333	1,72	0,1992
CC	0,0016547	1	0,0016547	0,33	0,5679
bloques	0,00108	2	0,00054	0,11	0,8974
Error total	0,164051	33	0,00497123		
Total (corr.)	0,79052	44			
